# Aspirin plus clopidogrel versus aspirin alone in patients with mild-to-moderate stroke: A systematic review and meta-analysis

**DOI:** 10.1097/MD.0000000000046821

**Published:** 2025-12-26

**Authors:** Mushood Ahmed, Zain Ali Nadeem, Areeba Ahsan, Eeshal Fatima, Laveeza Fatima, Rubab Zahra, Lilia Megherbi, Hritvik Jain, Rukesh Yadav, Muhammed Ameen Noushad, Tallal Mushtaq Hashmi, Raheel Ahmed, Shrey Gole, Kamleshun Ramphul

**Affiliations:** a Rawalpindi Medical University, Rawalpindi, Pakistan; b Allama Iqbal Medical College, Lahore, Pakistan; c Foundation University School of Health Sciences, Islamabad, Pakistan; d Services Institute of Medical Sciences, Lahore, Pakistan; e University hospitals Plymouth NHS trust, Plymouth, UK; f All India Institute of Medical Sciences (AIIMS), Jodhpur, India; g Maharajgunj Medical Campus, Institute of Medicine, Tribhuvan University, Kathmandu, Nepal; h Royal Brompton Hospital, London, UK; i National Heart and Lung Institute, Imperial College London, London, UK; j Stanford University, Stanford, CA; k Independent Researcher, Triolet, Mauritius.

**Keywords:** aspirin, clopidogrel, DAPT, ischemic stroke, mild-to-moderate stroke

## Abstract

**Background:**

Studies have shown that dual antiplatelet therapy (DAPT) is superior to aspirin monotherapy in patients with minor stroke or transient ischemic attacks. However, there is limited evidence regarding the efficacy and safety of DAPT in mild-to-moderate stroke.

**Methods:**

PubMed/MEDLINE, Embase, the Cochrane Library, and ClinicalTrials.gov were searched from inception till March 2024 for published randomized controlled trials and observational studies that compared aspirin plus clopidogrel versus aspirin monotherapy in patients with mild-to-moderate stroke. R version 4.3.2 was used to calculate risk ratios (RRs) with 95% confidence intervals (95% CIs).

**Results:**

A total of 4 studies reporting data for 15,173 patients were included. DAPT was associated with a non-significant trend of reduced risk of early neurological deterioration (END) (RR: 0.55, 95% CI: 0.28–1.05, *P* = .07) and recurrent ischemic stroke (RR: 0.65, 95% CI: 0.41–1.04, *P* = .07). The risk of recurrent hemorrhagic stroke (RR: 0.94, 95% CI: 0.47–1.86, *P* = .86), all-cause death (RR: 0.75, 95% CI: 0.52–1.08), or myocardial infarction (RR: 0.83, 95% CI: 0.45–1.54) was comparable across the two groups. DAPT was not associated with an increased risk of any bleeding event (RR: 0.70, 95% CI: 0.36–1.36).

**Conclusion:**

DAPT demonstrated a non-significant trend toward reduced risk of END and recurrent ischemic stroke without increasing the risk of bleeding events compared to aspirin monotherapy in patients with mild to moderate stroke. Further large-scale trials are needed to confirm these potential benefits.

## 1. Introduction

Acute ischemic stroke poses a significant global health challenge, with profound implications for morbidity and mortality worldwide. According to global statistics, stroke remains a leading cause of death and disability, with ischemic stroke accounting for the majority of cases.^[1–3]^ In the year 2019, the cumulative count of ischemic stroke-attributed fatalities amounted to 3.29 million, representing 50.3% of all stroke-related mortalities and 17.7% of the total deaths attributable to cardiovascular diseases.^[4–7]^ This underscores the critical significance of prioritizing preventive measures targeting ischemic stroke. Globally, individuals of both genders encounter an estimated lifetime risk of stroke at approximately 25%, commencing from the age of 25. However, regional disparities in stroke prevalence exist, with higher incidence rates observed in certain continents such as Asia, Africa, and Eastern Europe.^[8]^ Contributing factors include an aging population, rising prevalence of risk factors like hypertension and diabetes, and disparities in access to healthcare.^[9]^

The outcomes studied in the context of acute mild-to-moderate ischemic stroke carry significant clinical importance, directly impacting patient prognosis and quality of life. Recurrent stroke events, whether ischemic or hemorrhagic, can lead to further neurological deficits and increased disability, underscoring the need for effective secondary prevention strategies.^[10]^ Hemorrhagic complications, including intracranial hemorrhage, represent serious adverse events associated with antiplatelet therapy, posing a delicate balance between preventing ischemic events and avoiding hemorrhagic events.^[11]^

Current guidelines recommend aspirin monotherapy for secondary prevention of thrombotic events following mild-to-moderate stroke.^[12]^ However, to enhance efficacy, dual antiplatelet therapy (DAPT) with aspirin plus clopidogrel can be used. DAPT is commonly prescribed in patients with minor stroke or after percutaneous coronary intervention to minimize clot-related complications.^[13]^ Its duration varies based on the condition and individual factors. Previous meta-analyses have shown that the use of DAPT can lead to reduced risk of recurrent stroke in patients presenting with minor stroke or transient ischemic attacks.^[14–16]^ However, uncertainties persist regarding the comparative efficacy and safety of DAPT with aspirin plus clopidogrel versus aspirin alone in mild-to-moderate acute ischemic stroke. Addressing this gap is pivotal for refining treatment strategies and enhancing prognosis in acute mild-to-moderate stroke management. To our knowledge, this is the first systematic review and meta-analysis aimed at assessing the efficacy and safety of aspirin plus clopidogrel versus aspirin monotherapy in patients with mild-moderate acute ischemic stroke.

## 2. Methods

This systematic review and meta-analysis followed the guidelines established by the Preferred Reporting Items for Systematic Review and Meta-Analysis.^[17]^ The protocol of review is registered with PROSPERO (CRD42024526023). The Preferred Reporting Items for Systematic Review and Meta-Analysis checklist is provided as Table S1, Supplemental Digital Content, https://links.lww.com/MD/R14.

### 2.1. Data sources and search strategy

Two researchers (A.A. and R.Z.) independently searched PubMed/MEDLINE, Embase, the Cochrane Library, and ClinicalTrials.gov from inception to March 2024. No language restrictions were applied. The researchers manually examined references of retrieved randomized controlled trials (RCTs), observational studies, and prior reviews to ensure that all relevant articles were included. The search strategy used the following keywords and Medical Subject Headings terms: “aspirin,” “dual antiplatelet therapy,” “clopidogrel,” “ischemic stroke,” “mild to moderate stroke,” “non-minor stroke,” “intracranial embolism,” “randomized controlled trials,” and “observational studies.”

### 2.2. Eligibility criteria and outcomes

Studies were considered eligible for inclusion in our systematic review and meta-analysis if they: were published RCTs or observational studies comparing aspirin plus clopidogrel versus aspirin alone; included patients with non-cardiometabolic mild or moderate ischemic stroke or non-minor stroke as defined by the study inclusion criteria on the basis of National Institutes of Health Stroke Scale; therapy was started within 3 days; and evaluated at least one of the below-listed efficacy or safety outcome.

The efficacy outcomes included early neurological deterioration, recurrent ischemic stroke, recurrent hemorrhagic stroke, all-cause death, and myocardial infarction (MI). The safety outcome was the risk of any bleeding event.

### 2.3. Study selection and data extraction

The studies obtained from the literature search were imported to EndNote X9 (Clarivate Analytics), and duplicate records were removed. Two authors (A.A. and R.Z.) independently reviewed RCTs and observational studies based on titles and abstracts. This was followed by a review of the full texts of the articles. A third author (M.A.) was consulted in the event of any disagreements.

We extracted the following data from each eligible study: study/trial name, year of publication, study type, country, total sample size, inclusion criteria, dose of aspirin and clopidogrel in the treatment arm, dose of aspirin in the comparison arm, duration of DAPT, treatment onset time, duration of follow-up, age of patients, males, various risk factors/comorbidities such as hypertension, diabetes, smoking status, history of MI/ischemia, and any history of previous stroke or transient ischemic attack (TIA). We used a pre-piloted Excel sheet for data extraction.

### 2.4. Quality assessment of the included studies

Version 2 of the Cochrane Risk of Bias tool was used to assess the risk of bias for RCTs.^[18]^ Risk of bias was assessed across 5 domains: randomization, deviations from intended variation, missing outcome data, measurement of outcome, and selection of reported results. The trials were scored as high, with some concerns, or low risk of bias in each domain. The quality assessment of the observational studies was performed using the Cochrane Risk of Bias in Nonrandomized Studies – of Interventions (ROBINS-I) tool.^[19]^ The ROBINS-I tool uses seven domains to determine overall bias in each non-randomized clinical trial. Studies were classified as having low, moderate, serious, or critical risk of bias. Studies that had information missing in one or more domains were classified as NI (no information).

### 2.5. Statistical analysis

We conducted the meta-analysis on R version 4.3.2 using the packages “meta” and “metasens” via RStudio. The risk ratios (RRs) with 95% confidence intervals (CIs) were calculated using the Mantel-Haenszel method in a random effects model, which were presented in forest plots.^[20]^ We applied the Knapp-Hartung adjustment to the CIs.^[21]^ The Paule-Mandel estimator was used for tau^2^.^[22]^ Heterogeneity was assessed using the Cochrane Handbook of Systematic Reviews of Interventions arbitrary cutoff values for the Higgins *I*^2^ statistic, also considering the results of the Chi^2^ test: 0% to 40%, low heterogeneity; 30% to 60%, moderate heterogeneity; 50% to 90%, substantial heterogeneity; 75% to 100%, and considerable heterogeneity.^[23,24]^ We conducted an influence analysis by excluding one study at a time from the analyzed outcomes to explore the individual study effect on pooled effect sizes.

## 3. Results

### 3.1. Results of literature search

A systematic search of databases yielded 1734 records. The duplicate records were removed (n = 352). This was followed by the review of studies based on their titles and abstracts. A total of 37 studies were retrieved for the full-text assessment considering the eligibility criteria. Four studies met the inclusion criteria and were included in our meta-analysis. The study by Kim et al^[25]^ was not included in our review, as the researchers reported clinical outcomes for a similar cohort of patients, which was included in the propensity score matched analysis of Lee et al.^[26]^ The screening and study selection process is shown in Figure S1, Supplemental Digital Content, https://links.lww.com/MD/R14.

### 3.2. Baseline characteristics and bias assessment

Two of the included studies were RCTs^[27,28]^ and 2 were observational.^[26,29]^ The studies were published from 2014 to 2024 and reported data for 15,173 patients with mild-to-moderate stroke. Aspirin plus clopidogrel was administered in 7752 patients, while 7421 patients received aspirin monotherapy. RCTs^[27,28]^ reported the duration of DAPT (30 and 14 days). The DAPT was initiated within 48 hours of symptom onset in RCTs while in the observational study by Fan et al,^[29]^ treatment was initiated within 72 hours. Lee et al^[26]^ did not report the treatment onset time. The mean follow-up duration was 57.5 days, and the mean age of included patients ranged from 60 to 70.9 years The details of the relevant study and baseline characteristics are reported in Table 1 and Table S2, Supplemental Digital Content, https://links.lww.com/MD/R14.

**Table 1:** Baseline characteristics of the included studies and participants.

First author	Year	Country	Inclusion criteria	Sample size	Dose of DAPT	Dose of comparison	Duration of antiplatelet therapy	Treatment onset	Follow up
Yi et al	2014	China	Mild or Moderate IS (NIHSS score < 15)	DAPT = 284, Aspirin alone = 286	200 mg Aspirin + 75 mg clopidogrel daily from the d of administration to 30 d and 75 mg/d clopidogrel thereafter	Aspirin 200 mg/d until 30 d and 100 mg/d thereafter	30 d	<48 h	10 d
Lee et al	2020	Korea	Mild to Moderate acute (within 24 h onset) IS (NIHSS score < 10)	DAPT = 5591, Aspirin = 5591	NR	NR	NR	NR	90 d
Fan et al	2022	China	(1) acute mild-to-moderate IS, (2) within 72 h after the onset of IS, (3) features of acute IS on cerebral CT or MRI associated with the symptoms, (4) treatment with aspirin monotherapy or dual antiplatelet therapy with clopidogrel plus aspirin, and (5) medication with a high-intensity dose of statins with 40 mg of atorvastatin or 20 mg of rosuvastatin per d during hospitalization	DAPT = 375, Aspirin = 131	Clopidogrel at a loading dose of 300 mg or 75 mg on d 1, followed by a dose of 75 mg on day 2 through the hospitalization period	Aspirin at a physician-determined dose of 100 to 300 mg on the first d, followed by a dose of 100 mg per d on days 2 through the whole hospitalization period	NR	>72 h	40 d (days since hospitalization)
Chen et al	2024	China	Eligible patients were adults 18 yr and older with acute IS at the time of randomization (baseline NIHSS score 4–10; range from 0 to 42, with higher scores indicating greater stroke severity) who had been functioning independently (mRS scores ≤ 1; range from 0 [no symptoms] to 6 [death]) before a stroke and were enrolled up to 48 h after the onset of stroke symptoms (time last seen well)	DAPT = 1502, Aspirin = 1413	300 mg loading dose of clopidogrel plus aspirin, 100 mg; followed by clopidogrel, 75 mg/d, and aspirin, 100 mg/d, from day 2 to day 14 and clopidogrel, 75 mg/d, or aspirin, 100 mg/d, from d 15 to d 90	100 to 300 mg aspirin from d 1 to d 14, followed by aspirin, 100 mg/d, from d 15 to d 90	14	<48 h	90 d

C = Comparison (Aspirin Alone), CT = computed tomography, DAPT = dual antiplatelet therapy (Aspirin + Clopidogrel), END = early neurological deterioration, IS = ischemic stroke, MRI = magnetic resonance imaging, mRS = modified Ranking Scale, NIHSS = National Institutes of Health Stroke Scale, NR = not reported.

The quality assessment of the RCTs was performed using the Version 2 of the Cochrane Risk of Bias tool, which showed some concerns related to the randomization process in RCT by Yi et al,^[28]^ however, there was a low risk of bias in the other included RCT.^[27]^ The bias assessment of included observational studies showed moderate risk of bias. The details of bias assessment are reported in Figures S2–S5, Supplemental Digital Content, https://links.lww.com/MD/R14.

### 3.3. Results of the meta-analysis

#### 3.3.1. Efficacy outcomes

**Figure 1.**
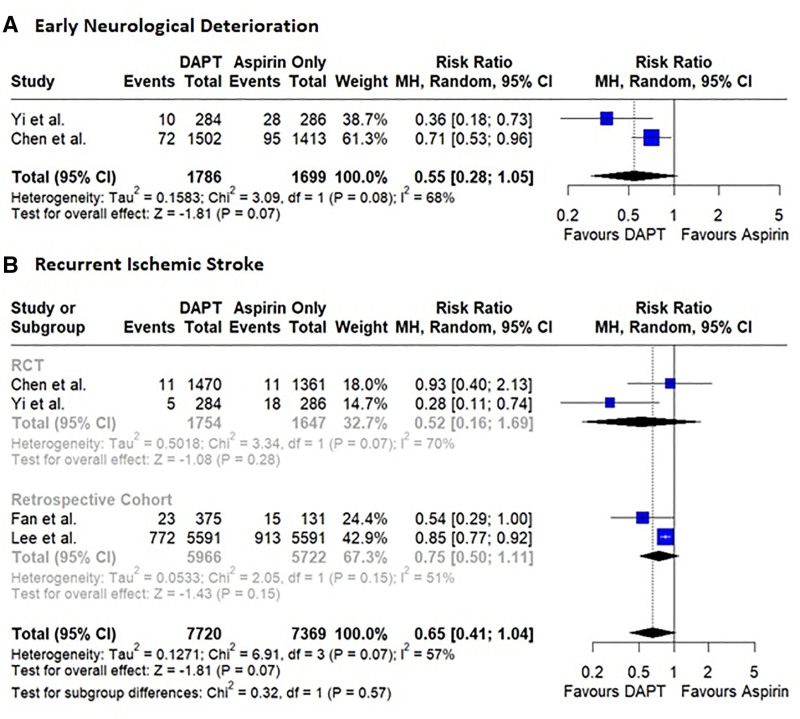
Forest plots showing pooled effect sizes for (A) Early neurological deterioration (B) Recurrent ischemic stroke. DAPT = dual antiplatelet therapy, RCTs = randomized controlled trials.

RCTs reported data for early neurological deterioration. DAPT was associated with a reduced risk of early neurological deterioration compared to aspirin alone without reaching statistical significance (4.5% END with DAPT vs 7.23% END with aspirin alone, RR: 0.55, 95% CI: 0.28–1.05, *P* = .07, *I*^2^ = 68%; Fig. 1A). We observed some evidence of publication bias (LFK index: −3.6, major asymmetry; Figure S6, Supplemental Digital Content, https://links.lww.com/MD/R14).

All 4 studies reported the occurrence of recurrent ischemic stroke. DAPT was associated with a reduced risk of recurrent ischemic stroke compared to aspirin alone, however, the results did not reach statistical significance (10.5% with DAPT vs 12.9% with aspirin monotherapy, RR: 0.65, 95% CI: 0.41–1.04, *P* = .07, *I*^2^ = 57%; Fig. 1B). Major asymmetry was observed (LFK index: −5.38, Figure S7, Supplemental Digital Content, https://links.lww.com/MD/R14).

**Figure 2.**
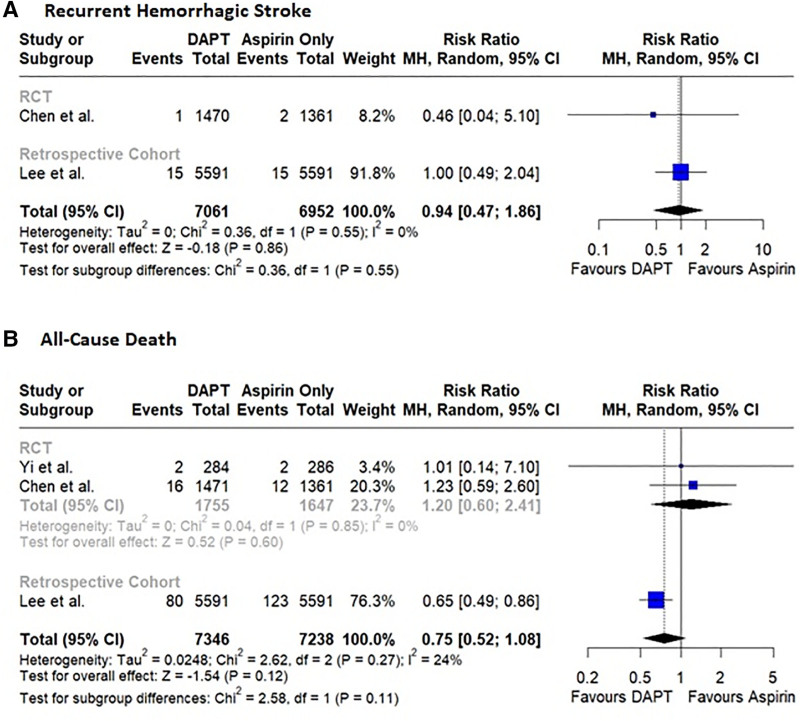
Forest plots showing pooled effect sizes for (A) Recurrent hemorrhagic stroke (B) All-cause death. DAPT = dual antiplatelet therapy, RCTs = randomized controlled trials.

Two studies reported the occurrence of recurrent hemorrhagic stroke. No significant difference was observed in the risk of recurrent hemorrhagic stroke between patients administered DAPT and patients administered aspirin (RR: 0.94, 95% CI: 0.47–1.86, *P* = .86, *I*^2^ = 0%; Fig. 2A). Major asymmetry was detected for publication bias (LFK index: −4.37, Figure S8, Supplemental Digital Content, https://links.lww.com/MD/R14).

Three studies reported the occurrence of all-cause death. No significant difference was observed in the risk of all-cause death between patients administered DAPT and patients administered aspirin (RR: 0.75, 95% CI: 0.52–1.08, *P* = .12, *I*^2^ = 24%; Fig. 2B), with some evidence of publication bias (LFK index: 4.5, major asymmetry; Figure S9, Supplemental Digital Content, https://links.lww.com/MD/R14).

**Figure 3.**
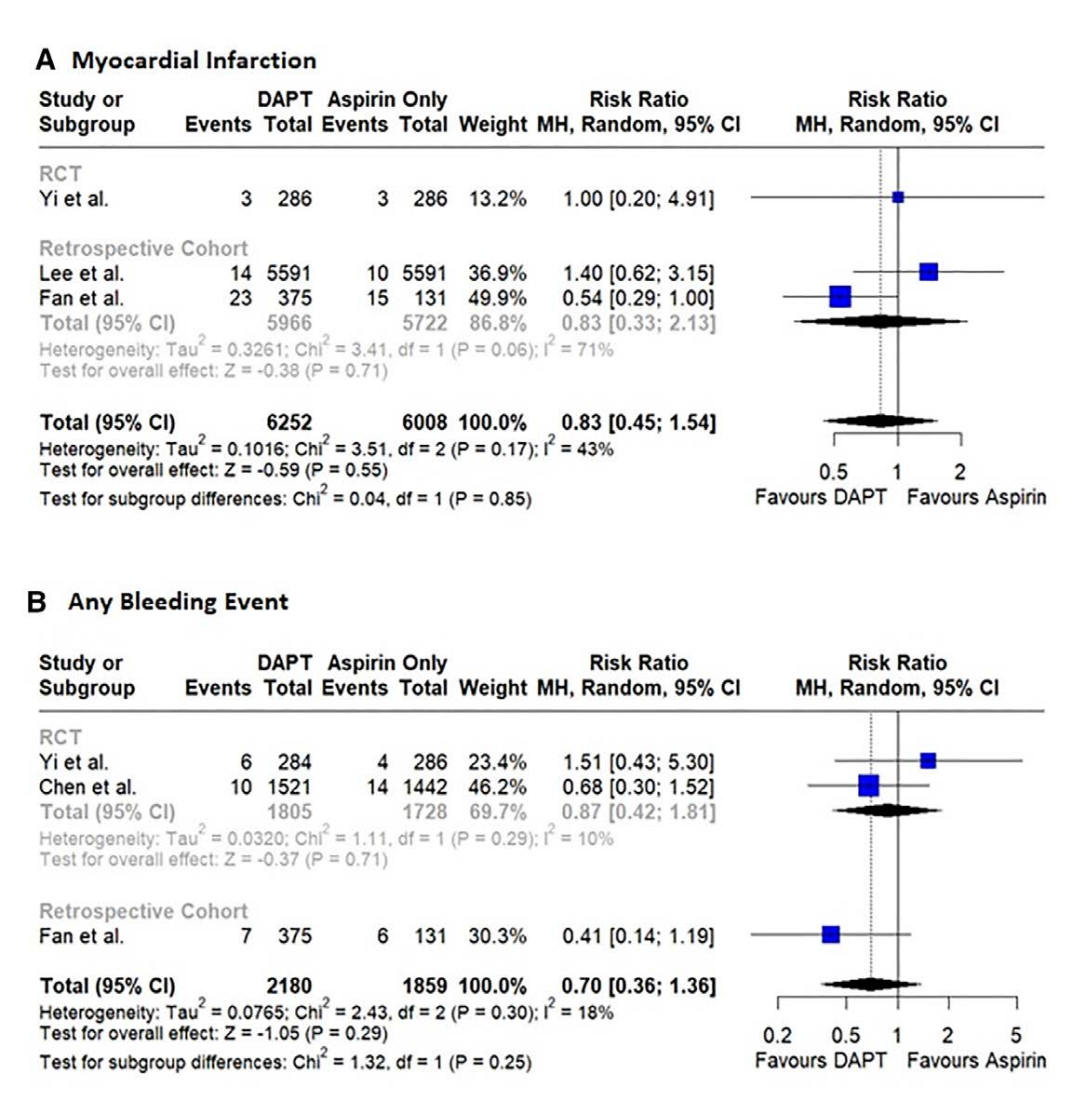
Forest plots showing pooled effect sizes for (A) Myocardial Infarction (B) Any bleeding event. DAPT = dual antiplatelet therapy, RCTs = randomized controlled trials.

Three studies reported the occurrence of MI. No significant difference was observed in the risk of myocardial infarction between patients administered DAPT and patients administered aspirin (RR: 0.83, 95% CI: 0.45–1.54, *P* = .55, *I*^2^ = 43%; Fig. 3A). There was no evidence of publication bias (LFK index: 0.21; Figure S10, Supplemental Digital Content, https://links.lww.com/MD/R14).

#### 3.3.2. Safety outcome

Three studies reported the occurrence of a bleeding event. No significant difference was observed in the risk of bleeding events between patients administered DAPT and patients who received aspirin only (RR: 0.70, 95% CI: 0.36–1.36, *P* = .29, *I*^2^ = 18%; Fig. 3B). Minor asymmetry was observed in the DOI plot (LFK index: 0.66, Figure S11, Supplemental Digital Content, https://links.lww.com/MD/R14).

The results of the sensitivity analysis are provided in Figures S12–S17, Supplemental Digital Content, https://links.lww.com/MD/R14.

## 4. Discussion

This comprehensive meta-analysis of 15,173 patients with mild to moderate stroke showed that DAPT with aspirin and clopidogrel reduced the risk of early neurological deterioration and recurrent ischemic stroke without reaching statistical significance. The risk of recurrent hemorrhagic stroke, all-cause death, and MI was nonsignificant across the 2 groups. The use of DAPT appeared safe, as the risk of bleeding events was comparable between the 2 groups.

Early neurological deterioration is associated with poor prognosis after ischemic stroke.^[30]^ The Antiplatelet Therapy in Acute Mild to Moderate Ischemic Stroke (ATAMIS) trial^[27]^ found that the use of aspirin and clopidogrel is superior to aspirin monotherapy for reducing the risk of early neurological deterioration. A randomized study by Yi et al^[28]^ also demonstrated that DAPT with aspirin plus clopidogrel was more effective in reducing the risk of early neurological deterioration. Current guidelines recommend aspirin monotherapy in patients with mild-to-moderate stroke^[12]^ however, the results of our meta-analysis show the superiority of DAPT in reducing the risk of early neurological deterioration and recurrent ischemic stroke. Moreover, the optimal use of tissue plasminogen activators remains an important predictor of improved outcomes in patients with stroke.^[31–36]^

A propensity score-matched analysis^[26]^ of Korean patients with mild-to-moderate stroke showed that the use of DAPT was associated with a significantly reduced risk of the composite outcome of recurrent stroke, MI, and all-cause mortality. It is the largest study to date that evaluated the use of aspirin plus clopidogrel in patients with mild-to-moderate stroke. A retrospective cohort study by Fan et al^[29]^ also showed that combination therapy with aspirin plus clopidogrel significantly reduced the risk of ischemic stroke, transient ischemic attack, and myocardial infarction. However, the Antiplatelet Therapy in Acute Mild to Moderate Ischemic Stroke trial (ATAMIS) trial found no significant difference between the two treatment groups for the incidence of recurrent stroke or all-cause death at a follow-up of 90 months. A possible explanation for these inconsistent findings is the short duration of DAPT (14 days) in the ATAMIS trial and the lack of statistical power in the secondary outcomes. In the future, RCTs that are designed to evaluate vascular events (new stroke, MI, and all-cause death) as primary outcomes are required to better understand the efficacy of DAPT in patients with mild-to-moderate stroke.

The risk of bleeding with DAPT is an important concern. The Intensive Statin and Antiplatelet Therapy for Acute High-Risk Intracranial or Extracranial Atherosclerosis and the Platelet-Oriented Inhibition in New TIA and Minor Ischemic Stroke trials conducted in patients with minor stroke found that DAPT with aspirin plus clopidogrel resulted in an increased risk of moderate to severe bleeding.^[37,38]^ However, our meta-analysis showed that the use of DAPT was safe in mild-to-moderate stroke and not associated with hemorrhagic complications. This can be attributed to the short duration of DAPT in the ATAMIS trial as compared to the Intensive Statin and Antiplatelet Therapy for Acute High-Risk Intracranial or Extracranial Atherosclerosis and Platelet-Oriented Inhibition in New TIA and Minor Ischemic Stroke trials (14 days vs 21 days and 90 days, respectively). Two of the included studies were observational in nature, which introduces the possibility of residual confounding despite statistical adjustment. Our ROBINS-I assessments indicated a moderate risk of bias for these studies, mainly due to potential selection bias and unmeasured confounders. Although their findings were generally consistent with those of RCTs, the inclusion of observational data may have influenced the overall effect estimates. Therefore, our results should be interpreted with caution. Moreover, the study by Lee et al^[26]^ in our pooled analysis did not mention the duration and dosage of DAPT as it was a registry-based retrospective cohort study. The findings of our meta-analysis should be considered hypothesis-generating, and the evidence of prospectively designed studies will better help the clinicians regarding the safety of DAPT in mild-to-moderate stroke.

### 4.1. Clinical implications

The findings of this meta-analysis suggest that dual antiplatelet therapy with aspirin and clopidogrel may provide added protection against early neurological deterioration and recurrent ischemic stroke in patients with mild-to-moderate stroke, without a clear increase in bleeding risk. Although the results did not reach statistical significance, the consistent direction of effect across studies highlights a potential benefit that could inform clinical decision-making, especially in patients considered at higher risk for early progression. At the same time, the uncertainty of evidence underscores the need for cautious application in practice, with individualized risk–benefit assessment until stronger evidence is available.

### 4.2. Future research directions

Future RCTs are needed to validate these findings, ideally with larger, more diverse populations and longer follow-up periods to better capture vascular outcomes such as recurrent stroke, myocardial infarction, and mortality. Trials designed with varying durations of DAPT could also clarify the optimal treatment window that balances efficacy with safety. Moreover, including patients from different racial and geographic backgrounds will be critical to improving the generalizability of results, as most existing evidence is derived from Asian cohorts. Well-powered studies focusing on safety endpoints will be particularly important to confirm that DAPT does not increase bleeding risk in this population.

### 4.3. Strengths and limitations

The meta-analyses^[14,15,39,40]^ conducted in the past have evaluated the role of DAPT with aspirin and clopidogrel/ticagrelor in patients with minor stroke or transient ischemic attack. However, there was a lack of evidence regarding the efficacy and safety of aspirin plus clopidogrel compared to aspirin monotherapy in mild-to-moderate stroke. To our knowledge, this is the first meta-analysis that evaluated the efficacy and safety of combination therapy with aspirin plus clopidogrel in patients with mild-to-moderate stroke.

Our study has some limitations as well. The inclusion criteria and the baseline National Institutes of Health Stroke Scale scores for patients varied across the included studies as the grading system used for ranking stroke severity is currently arbitrary. The studies included in our meta-analysis enrolled Chinese and Korean patients. Studies with diverse patient populations are required to confirm the generalizability of our findings in other racial groups. Two of the included studies were observational, and the treatment selection was based on the decision of physicians rather than randomization. However, we attempted to overcome this by performing subgroup analysis to report pooled effect estimates for RCTs and observational studies separately. We included propensity score-matched data from the study by Lee et al^[26]^ to adjust for the baseline imbalances between two treatment groups however the risk of residual confounding cannot be ignored. The safety outcomes could not be assessed extensively due to limited available data. Although our search strategy was comprehensive and included four major databases (PubMed/MEDLINE, Embase, the Cochrane Library, and ClinicalTrials.gov), only four eligible studies were identified. While this may limit the robustness of the pooled conclusions, the approach was consistent with Cochrane recommendations and aligned with our PROSPERO-registered protocol, which prespecified the databases to be searched. Another important limitation is that the included studies did not report outcomes stratified by race or sex. As stroke risk and response to therapy are known to vary across racial and ethnic groups, as well as between men and women, the lack of demographic data limits the generalizability of our findings. Factors such as pregnancy, hormonal therapies, and other sex-specific variables may also influence outcomes but could not be assessed in this analysis. Future trials should ensure adequate representation of diverse racial and sex groups and report stratified outcomes to improve the inclusiveness and applicability of treatment strategies.

## 5. Conclusion

DAPT with aspirin plus clopidogrel showed a nonsignificant trend toward reducing the risk of END and recurrent ischemic stroke. DAPT appeared to be safe, as the risk of any bleeding events was comparable across the 2 groups. However, evidence from additional RCTs in diverse racial groups is required to confirm these potential benefits.

## Author contributions

**Conceptualization:** Mushood Ahmed, Shrey Gole, Kamleshun Ramphul.

**Data curation:** Mushood Ahmed, Lilia Megherbi, Shrey Gole, Kamleshun Ramphul.

**Formal analysis:** Zain Ali Nadeem, Areeba Ahsan, Eeshal Fatima, Rubab Zahra, Hritvik Jain.

**Methodology:** Zain Ali Nadeem, Areeba Ahsan, Eeshal Fatima, Rubab Zahra, Hritvik Jain.

**Project administration:** Mushood Ahmed, Muhammed Ameen Noushad, Shrey Gole, Kamleshun Ramphul.

**Software:** Zain Ali Nadeem, Areeba Ahsan, Eeshal Fatima, Rubab Zahra, Hritvik Jain.

**Supervision:** Mushood Ahmed, Raheel Ahmed.

**Validation:** Mushood Ahmed, Raheel Ahmed, Shrey Gole, Kamleshun Ramphul.

**Visualization:** Mushood Ahmed, Raheel Ahmed, Shrey Gole, Kamleshun Ramphul.

**Writing – original draft:** Mushood Ahmed, Zain Ali Nadeem, Areeba Ahsan, Laveeza Fatima, Lilia Megherbi, Rukesh Yadav, Tallal Mushtaq Hashmi, Raheel Ahmed.

**Writing – review & editing:** Laveeza Fatima, Lilia Megherbi, Hritvik Jain, Rukesh Yadav, Muhammed Ameen Noushad, Tallal Mushtaq Hashmi, Raheel Ahmed, Shrey Gole.

## Supplementary Material


